# The Prevalence of Aflatoxinogenic *Aspergillus parasiticus* in Jordan

**DOI:** 10.1155/2012/675361

**Published:** 2012-04-23

**Authors:** Nisreen Al-Hmoud, Mohammed A. Ibrahim, Hiyam Al-Rousan, Abbas Alseyah

**Affiliations:** ^1^Biosafety Unit, Royal Scientific Society, P.O. Box 1438, Amman 11941, Jordan; ^2^Environmet Management Department, Princess Sumaya University for Technology, P.O. Box 1438, Amman 11941, Jordan

## Abstract

Aflatoxins are potent carcinogens and produced by almost all *Aspergillus parasiticus* isolates and about 35% of *Aspergillus flavus* isolates. Chemical methods are used for detection of aflatoxins in food and feed. These methods cannot detect aflatoxinogenic fungi in samples, which contain undetectable amounts of aflatoxins. The objective of this research work was to ascertain the importance of molecular and microbiological methods in detection of aflatoxinogenic fungus *A. parasiticus* in food and feed samples in Jordan. Specific media for the detection of aflatoxins showed the prevalence of *A. parasiticus* (6–22%) in contaminated food and feed samples. HPLC method confirmed the presence of aflatoxins B1, B2, G1, and G2 in food sample contaminated with *A. parasiticus*. Primer set OmtBII-F and OmtBII-R amplified DNA fragment of 611 base pairs from genomic DNA of aflatoxinogenic *A. parasiticus* isolated from food and feed samples but could not amplify DNA fragment of nonaflatoxinogenic *A. flavus*. The results of this study showed the prevalence of aflatoxinogenic *A. parasiticus* in food and feed samples in Jordan and give further evidence of suitability of microbiological and molecular methods in detection of aflatoxins, which are reliable low-cost approach to determine food and feed biosafety.

## 1. Introduction

Aflatoxins are fungal metabolites known for their potent carcinogenic properties. The ability of aflatoxin production has been reported in various species of the *Aspergillus *genus, inside and outside the Flavi group [1]. However, various studies have indicated that aflatoxins are primarily produced by *Aspergillus parasiticus* and *Aspergillus flavus* isolates. Additionally, these studies indicated that the majority of *A. flavus* isolates (60–70%) are atoxigenic [1–3], whereas almost all isolates of *A. parasiticus* are aflatoxinogenic and are potential aflatoxins producers in agricultural commodities [3–5].

Chemical methods are used for detection of aflatoxins in food and feed. These methods cannot detect aflatoxinogenic fungi in contaminated samples, which contain undetectable amounts of aflatoxins. On the other hand, microbiological and molecular detection methods have been used to ascertain the aflatoxinogenicity of *A. flavus and A. parasiticus*. During the last two decades, Polymerase Chain Reaction (PCR) methods have been developed for detection of aflatoxigenic fungi. In this respect, several primers have been designed for several genes in the biosynthetic pathways of aflatoxins; for example, afl, nor, omt, ord, tub, ver, [1, 5–8]. Microbiological methods were found powerful in detection of aflatoxinogenic fungi. *A. parasiticus* colonies showed beige rings when are grown in specific aflatoxins detection media, these rings surrounding fungal colonies were confirmed as indicators of aflatoxins production [9]. The objective of the present investigation was to ascertain the prevalence of *A. parasiticus* in food and feed samples in Jordan by using chemical, molecular, and microbiological methods.

## 2. Materials and Methods

### 2.1. Fungal Strains

*A. parasiticus* BS23 (a producer of aflatoxins) and *A. flavus* BS48 (a non producer of aflatoxins) were obtained from the fungal strain collection at the Biosafety Unit, Royal Scientific Society. These strains were used as standard strains for microbiological and molecular experiments.

### 2.2. Sample Collection and Isolation of Fungi

Samples of food and feed were collected from the local markets in Amman, Jordan over a period of 23 months from January 2010 to November 2011. Fungi were isolated from food and feed following the Food and Agricultural Organization (FAO) standard methods [10]. Potato Dextrose Agar (PDA) plates were inoculated using decimal dilutions of the test sample. The plates were aerobically incubated at 25°C for 5 days. The numbers of colony-forming units (CFU) of moulds per gram of product were calculated from the number of colonies obtained on plates chosen at dilution levels [10].

### 2.3. Morphological Characterization

Specified quantities of fungal spores suspension were inoculated on 9 cm diameter Petri dishes containing 20 mL of CZ (Sucrose 30 g/L, K_2_HPO_4_ 1 g/L, NaNO_3_ 2 g/L, KCl 0.5 g/L, MgSO_4_·7H_2_O 0.5 g/L, FeSO_4_·7H_2_O 0.01 g/L, ZnSO_4_·7H_2_O 0.01 g/L, CuSO_4_·5H_2_O 0.005 g/L, Agar 20 g/L). Cultures were incubated for 14 days, in the dark, at 25°C and then morphological characterisitics of fungal isolates were analysed according to Klich, 2002 [11]. The colony colour and conidia morphology were investigated.

### 2.4. Aflatoxins Detection

#### 2.4.1. Microbiological Methods

The production of aflatoxin by *A. parasiticus* and *A. flavus* was ascertained following the method reported by Jaimez Ordaz et al. [9]. Yeast extract sucrose agar (YES) and YCSD consisted of YES supplemented with 0.3% cyclodextrin and 0.6% sodium deoxycholate that were used for detection of aflatoxins production ability of *A. parasiticus* and *A. flavus*. The aflatoxinogenic fungal isolate forms a beige ring surrounding the colony which is visible without need of UV light (365 nm) exposure; but the ring is not observed for any of the nonaflatoxinogenic fungal isolates. Furthermore, it is possible to visualize the blue fluorescent ring surrounding aflatoxinogenic colonies under UV light [9]. 

#### 2.4.2. HPLC Method

Determination of aflatoxins was conducted in the laboratories of Biosafety Unit of Royal Scientific Society, Amman, Jordan according to AOAC official method 990.33. In brief, the test sample was finely ground, extracted with methanol followed by 0.1 M HCl and filtered. The filtrate was mixed with 10% NaCl solution followed by the addition of hexane. Then aqueous layer was mixed with CH_2_Cl_2_ and the eluate of CH_2_Cl_2_ was collected and evaporated in steam bath under gentle stream of nitrogen. The purified sample was then derivatized with trifluoroacetic acid. Aflatoxins (B1, B2, G1, and G2) were separated by reversed-phase liquid chromatography and detected by fluorescence. Fluorescence detector was operated at 360 nm excitation filter and 440 nm emission filter. HPLC system (Shimadzu 20A) was used to perform the test.

### 2.5. Molecular Experiments

#### 2.5.1. Extraction of Genomic DNA

The genomic DNA was extracted from mycelia of fungal isolates obtained from 7 days old cultures grown in YES liquid media. The mycelia were frozen and grounded into a fine powder in liquid nitrogen and DNA was extracted by the Qiagen DNeasy Plant Mini-Kit. The concentration and purity of extracted DNA were determined according to the reported methods [12, 13].

#### 2.5.2. Primers

The primers and their sequences (Table 1) which were used in the PCR amplification experiments were reported in previous work [1, 5, 14]. The primers were obtained from Alpha DNA/Canada.

#### 2.5.3. DNA Amplifications Conditions

The reported PCR amplification conditions for amplification of DNA fragments specified by OmtBII and Nor1 primer pairs were conducted according to Rahimi et al., 2008 [5] and Criseo et al., 2001 [7], respectively.

#### 2.5.4. Gel Electrophoresis

The amplified DNA fragments and DNA marker ladder of 100 bp (Qiagen) were separated using 1.5% agarose gel and visualized under UV light after staining with ethidium bromide for molecular size determinations in base pair (bp) of DNA fragments [15].

## 3. Results

### 3.1. Prevalence of Fungi in Food and Feed Samples

A total of forty seven samples of food and feed commercially available in Amman, Jordan were obtained during the period from January 2010 to November 2011. The incidence of fungi in the food and feed samples as determined by CFU/gram showed wide variations that were in the range of 0.2∗10^2^–2.4∗10^4^ CFU/gram (Table 2).

### 3.2. Microbiological Characterization of Aflatoxinogenic Fungi

**Figure 1:**
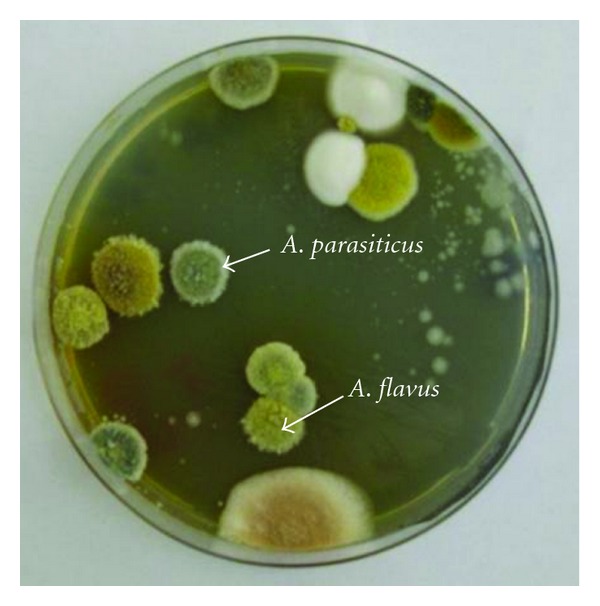
*A. flavus* and *A. parasiticus* colonies grown on CZ plate isolated from nuts sample.

In the present study, it was possible to identify two species that belong to genus *Aspergillus* according to colony color on CZ and conidia morphology. The two morphological characteristics were considered for morphological characterization of fungal isolates. Isolates which had shown dark-green colonies and rough conidia were classified as *A. parasiticus*. However, the other isolates showed the morphological characteristics of *A. flavus* with yellowish-green colonies and smooth conidia (Figure 1). The identified isolates of each species which were recovered from food and feed samples were subjected for further microbiological characterization for detection of aflatoxinogenic fungi by using specific aflatoxin detection media (YES and YCSD). It was possible to observe a beige ring surrounding *A. parasiticus* colonies when grown on YES and YCSD media. This observation indicated that the colonies are aflatoxinogenic. The ring was visible with and without UV light for *A. parasiticus* colonies but was not observed for any of tested *A. flavus* isolates. The results also showed that the aflatoxins producing isolates (*A. parasiticus*) are more prevalent in nuts and feed samples as compared with other samples (Table 2). 

### 3.3. HPLC Method

**Figure 2:**
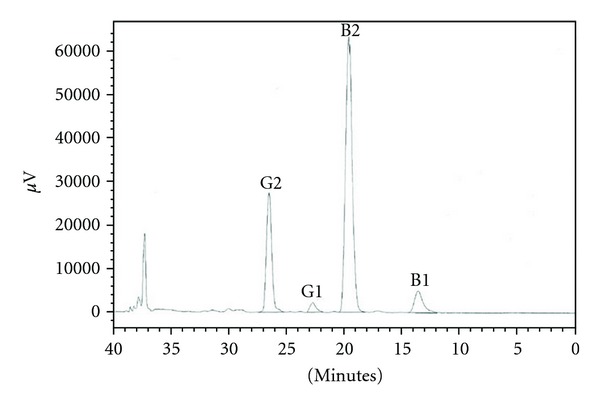
HPLC chromatogram of aflatoxins (B1, B2, G1, and G2) obtained from nuts sample contaminated by *A. parasiticus*.

A chromatogram obtained in this study for aflatoxins detected by HPLC method is shown in Figure 2. The results demonstrated the occurrence of four aflatoxins (B1, B2, G1, and G2) in sample of nuts contaminated with *A. parasiticus*. On the other hand, the tested samples contaminated with *A. flavus* showed undetectable amounts of aflatoxins. In this study, twenty two food and feed samples were investigated for the presence of aflatoxins by HPLC method; the obtained results confirmed that all tested samples contained ≤4 ppb total aflatoxins. The tested samples were nuts, wheat flour, maize, sun flower, coffee beans, and feed. 

### 3.4. Molecular Genomic Analysis

**Figure 3:**
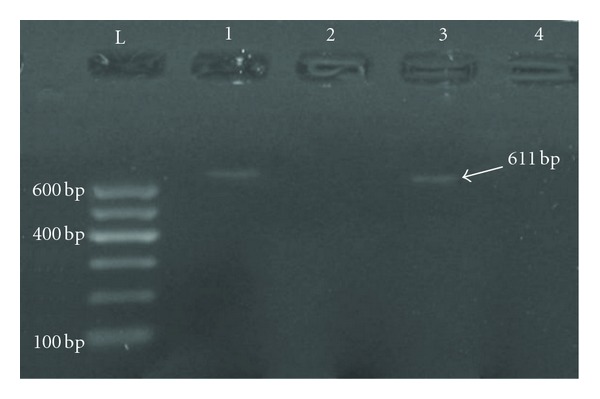
Detection of PCR amplified OmtBII sequence (611 bp) in the reference aflatoxins producing strain *A. parasiticus *BS23. Lane L indicates the 100 base pair ladder, Lane 1 and Lane 3 represent aflatoxinogenic *A. parasiticus* BS23, Lane 2 represents *A. flavus* BS48 (a nonproducer of aflatoxins), and Lane 4 represents PCR negative control. Electrophoresis was performed on 1.5% agarose gel and run with 3 volt cm^−1^.

**Figure 4:**
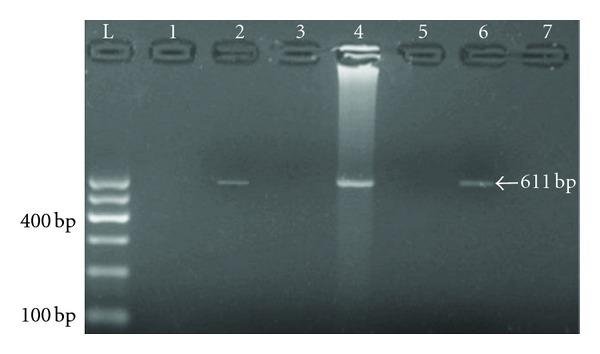
PCR amplified OmtBII sequence (611 bp) in three *A. parasiticus *isolates obtained in this study. Lane L indicates the 100 base pair ladder, Lane 1 represents PCR negative control, Lane 2, 4, and 6 represent three *A. parasiticus *isolates, and lanes 3, 5, and 7 represent the three *A. flavus* isolates. Electrophoresis was performed on 1.5% agarose gel and run with 3 volt cm^−1^.

Differences were observed in the concentrations and purities of extracted genomic DNA of fungal mycelia obtained from tested food and feed samples. The lowest yield of extracted DNA was 37 *μ*g/mL, whereas the highest yield was 57 *μ*g/mL. The purity of the extracted DNA showed variations between 1.57 and 1.81. The results of PCR experiments for the detection of amplified DNA fragments specified by the tested primer pairs (OmtBII-F and OmtBII-R) showed the presence of 611 bp amplified fragments in genomic DNA extracted from *A. parasiticus* BS23 (a producer of aflatoxins), this band was not observed in the genomic DNA of *A. flavus* BS48 (Figure 3). The 611 bp DNA fragment was also observed in other three tested isolates of *A. parasiticus *recovered from contaminated food samples. On the other hand, no bands were detected which represent the amplified 611 bp DNA fragment from the other three tested *A. flavus* isolates (Figure 4). The set of Nor1 primers gave negative results.

## 4. Discussion 

Aflatoxins are secondary carcinogenic metabolic products produced primarily by two fungal species, *A. flavus *and *A. parasiticus *[16]. The result of this study showed that food sample contaminated with *A. parasiticus* was a producer of four types of aflatoxins (B1, B2, G1, and G2). Aflatoxin B1 is considered the most toxic and is produced by both *A. flavus and A. parasiticus*, whereas Aflatoxins G1 and G2 are produced exclusively by *A. parasiticus* [16]. One important argument of this study was to ascertain the validity of microbiological and molecular tests for detection of aflatoxins. Microbiological tests were found suitable for detection of aflatoxins produced by *A. parasiticus*. It was possible to observe a beige ring surrounding *A. parasiticus* colonies when grown in aflatoxins detection media (YES and YCSD). Jaimez Ordaz et al. [9] were able to show that beige rings, which were observed surrounding fungal colonies, were indicators of aflatoxins production. These rings were visible without the need for exposure to UV light. Furthermore, the investigators showed that the rings were not observed for any of the nonaflatoxinogenic strains. Moreover, the researchers were able to confirm by HPLC analysis the aflatoxinogenic nature of fungal isolates which were detected by microbiological methods [9]. The results reported in this study indicated that microbiological tests are reliable for detection of aflatoxinogenic fungi. The beige ring was visible with and without UV light for aflatoxinogenic *A. parasiticus* colonies but was not observed for any of the tested nonaflatoxinogenic *A. flavus* isolates. Thus, it is possible to suggest the usefulness of this noncostly method for detection of aflatoxinogenic fungi in contaminated food and feed samples in comparison with HPLC method. Furthermore, recent advances in molecular analysis indicated and confirmed the possibility of using PCR methods for detection of contamination of food and feed with aflatoxinogenic fungi at shorter time and with high confidence. The results of this study also showed that the *A. parasiticus* had the DNA sequence of the omt gene, which could be amplified by suitable PCR primers (OmtBII). The same set of primers could not amplify the DNA sequence of genomic DNA obtained from *A. flavus*. These results confirm earlier reported results about the suitability of the DNA sequence of the omt gene for design of specific primers for detection of aflatoxinogenic fungi [1, 5, 8]. 

It is noteworthy to mention that the production of aflatoxins by aflatoxinogenic fungi requires certain environmental conditions [9, 17]. Therefore, food and feed samples which do not contain aflatoxins but are contaminated with aflatoxinogenic fungi might be vulnerable to aflatoxins contamination when environmental conditions during storage are suitable for aflatoxins production. 

The significance of this study also comes from reported results which indicate that about 25% of world's supply of food is contaminated with mycotoxins and aflatoxins causing most serious health problem [18]. Moreover, there are other reports which have emphasized on *A. parasiticus* as the most important aflatoxins-producing species that contaminate foodstuffs and beverages for human consumption and the necessity for the development of new specific highly sensitive PCR assays for detection [18–20]. 

At the end of this discussion, we would like to highlight another important issue in regard of food or feed contamination with aflatoxins. The allowed amounts of sum total of aflatoxins (B1, B2, G1, and G2) in crops such as nuts, groundnuts, grains, and dried fruits are within the range of 4.0 and 15.0 *μ*g/kg in the European Union [21] and <20 ppb (*μ*g/kg) in the United States [22] and the Hashemite Kingdom of Jordan [23]. In the present investigation, the detected amounts of total aflatoxins in tested food samples were well below 20 ppb. However, it is important to note that the long-term chronic exposure to low amounts of aflatoxins can increase the risk of liver cancer [24, 25]. 

## 5. Conclusions 

This study provides for first time useful information on the prevalence of *A. parasiticus* in food and feed in Jordan. Contaminated food samples with *A. parasiticus* contained the four types of aflatoxins (B1, B2, G1, and G2). The high incidence of *A. parasiticus* in contaminated food and feed emphasizes the need for using rapid, low-cost, and reliable microbiological and molecular methods for detection of this fungus. The study gives further evidence in support of microbiological and molecular detection methods of aflatoxins as low-cost methods as compared with HPLC methods for continuous monitoring of food and feed products. 

## Figures and Tables

**Table 1:** The primer sequences of target genes and expected product length in base pairs (bp) for PCR amplified DNA fragments.

Primer pair	Gene primer	Sequence (5′→3′)	PCR product l size (bp)	References
Nor1-F Nor1-R	aflD	ACC GCT ACG CCG GCA CTC TCG GCA C GTT GGC CGC CAG CTT CGA CAC TCC G	400	[1, 14]
OmtBII-F OmtBII-R	omtB	ATG TGC TTG GGI TGC TGTG G GGA TGT GGT YAT GCG ATT GAG	611	[5]

**Table 2:** Fungi detected in contaminated food and feed samples analyzed during January 2010 to November 2011.

Types of samples	CFU/g	*A. flavus* (%)	*A. parasiticus* (%)	Other fungi (%)
Nuts (pistachio, cashew, almonds, and peanuts)	8.3∗10^2^–1.0∗10^4^	18	22	65
Maize	1.0∗10^2^–1.5∗10^3^	7	8	85
Sun flower	(0.3–0.9)∗10^2^	—	—	—
Coffee beans	0.2∗10^2^–1.0∗10^3^	—	—	—
Wheat flour	0.8∗10^2^–2.4∗10^4^	4	6	90
Feed	1.4∗10^3^–1.1∗10^4^	16	20	64
